# Encouraging physicians' continuous knowledge-sharing in online health communities: A motivational perspective

**DOI:** 10.3389/fpubh.2022.1036366

**Published:** 2022-11-07

**Authors:** Xin Zhang, Shanzhen Gao, Yanyan Cheng, Fanbo Meng

**Affiliations:** ^1^Management School, Tianjin Normal University, Tianjin, China; ^2^School of Business, Jiangnan University, Wuxi, China

**Keywords:** online health community, practical benefit, psychological reward, perceived connectedness, online seniority status, continuous knowledge-sharing

## Abstract

Online health communities (OHCs) as an essential means of patient education can significantly improve patients' health literacy and treatment outcomes. However, sustaining these social benefits brought by OHCs establishes the prerequisite that physicians can continuously share their knowledge on OHCs. Although previous studies have explored physicians' knowledge-sharing in OHCs, scholarly knowledge related to the means of motivating physicians to continue sharing their knowledge remains limited. Therefore, this study developed a research model based on motivation theory to explore the influence of practical benefits, psychological rewards, and perceived connectedness with OHCs on physicians' continuous knowledge-sharing behaviors and the contingent role of physicians' online seniority status. The research model and relevant hypotheses were examined using objective data from one of the leading OHCs in China. The empirical results reveal that both practical benefits and psychological rewards positively affect physicians' continuous knowledge-sharing behaviors. However, an unexpected finding is that perceived connectedness is negatively associated with physicians' continuous knowledge-sharing behaviors. In addition, physicians' online seniority status strengthens the relationship between practical benefits and continuous knowledge-sharing behaviors but weaken the role of psychological rewards and perceived connectedness on continuous knowledge-sharing behaviors. This study contributes to the understanding of the motivational mechanisms underlying physicians' continuous knowledge-sharing behaviors in OHCs and provides significant practical implications for practitioners of OHCs.

## Introduction

Online health communities (OHCs) refer to web-based platforms that provide all segments of the population with freely accessible health information and services for improving patients' health outcomes and reducing the health disparity between urban and rural residents (1–3). As a significant complement to traditional health services, OHCs allow physicians to share free health knowledge that promotes patient education and provides social support for patients (4, 5). However, a major challenge in the sustainable development of OHCs is physicians' under-contribution (6–8) since the sustainability of OHCs significantly relies on all members' active participation, especially physicians' continuously sharing free health-related knowledge (9, 10). The lack of such contributions on the part of physicians threatens to weaken the real value of OHCs (11). In other words, an OHC's ability to motivate physicians to persistently contribute their knowledge to the online platform is likely to determine its competitive advantages. Therefore, examining the motivational factors of physicians' continuous knowledge-sharing behaviors in OHCs is essential.

Abundant prior research has extended the scholarly understanding of factors influencing physicians' participation and knowledge-sharing behaviors in OHCs. These motivators can be classified in terms of two main perspectives: practical benefits and psychological rewards. The former term denotes the tangible rewards that physicians receive from their participation in the network, while the latter represents the non-tangible benefits that physicians receive from their participation. To be more specific, the goal of physician participation is to obtain enjoyment and satisfaction. From the perspective of practical benefits, the associated motivators include financial incentives (12, 13) and online reputation (14–16). In contrast, motives relating to psychological rewards consist of enjoyment in helping others (14) and psychological satisfaction (13, 17). Furthermore, since OHCs incorporate features of both online relational communities and online transactional communities, physicians have developed their social networks to some extent through their connections with OHCs (18). Individuals who feel highly connected with a virtual community are more likely to have a positive view of that community and contribute more to it (19). According to the work of Chou et al. (20), we may postulate that when physicians have a higher level of perceived connectedness with an OHC, they will maintain a long-lasting relationship with it and continuously contribute knowledge, thus improving the sustainability of OHCs. However, to our best knowledge, the role of physicians' perceived connectedness as it relates to their continuous knowledge-sharing remains obscure, and the combined effects of practical benefits, psychological rewards, and perceived connectedness on physicians' continuous knowledge-sharing in OHCs have received scant examination. Based on these considerations, therefore, the first research question of this study is as follows: *How do practical benefits, psychological rewards, and perceived connectedness jointly motivate physicians' continuous knowledge-sharing behaviors in OHCs?*

Besides the above-mentioned motivators, physicians' characteristics (e.g., seniority status) play a critical moderating role in shaping the relationship between motivators and knowledge-sharing. Previous research has found physicians' offline professional status to relate positively to their prestige and financial status and identified this status as a primary characteristic influencing physicians' behavior in OHCs (18). Physicians with higher offline seniority status (i.e., professional titles in hospitals) are highly motivated by reputation and psychological rewards and less motivated by monetary rewards related to participating in and contributing their knowledge to OHCs (13, 18, 21). However, compared to this offline seniority status, physicians' online seniority status (honorary title) in OHCs has drawn less academic attention in previous investigations examining physicians' knowledge-sharing, particularly in the context of continuous knowledge-sharing. Physicians' online seniority status, as indicated by a platform-generated honorary title, also reflects patients' high-tier recognition of and trust in their healthcare service quality in the online context (22). However, different from physicians with higher offline status, those with a higher online honorary title may reveal varying levels of response in the form of continuous knowledge-sharing behaviors when they are motivated by practical benefits, psychological rewards, or perceived connectedness with OHCs. Therefore, it is necessary to investigate how physicians' online seniority status (honorary title in OHCs) shapes the relationship between the various motivators and continuous knowledge-sharing. Based on the above discussion, another research question for this study is: *Are the effects of practical benefits, psychological rewards, and perceived connectedness on physicians' continuous knowledge-sharing behaviors contingent on physicians' online seniority status?*

To address the above two research questions, this study draws upon motivation theory and the relevant literature on knowledge-sharing in OHCs to develop a research model and related hypotheses. Next, we collected data from the “Good Doctor Online” website (www.haodf.com) to test our hypotheses. This study makes three contributions. From the perspective of the sustainability of OHCs, this study is one of the first to investigate the motivational mechanism of physicians' continuous knowledge-sharing behaviors that previous studies have significantly overlooked. As its second contribution, this paper provides additional insight into the factors that motivate physicians to continuously share knowledge in OHCs through examining the integrated effect of practical benefits, psychological rewards, and perceived connectedness on continuous knowledge-sharing behaviors. Another key contribution of this study is its examination of the contingent role of a physician's online seniority status, as generated by the OHC where he or she is participating, in terms of the relationship between three-dimensional motivators and continuous knowledge-sharing, which further enriches the existing literature regarding the motivational factors behind physicians' knowledge-sharing in OHCs.

The remainder of this paper is organized as follows. First, we discuss the existing literature on physicians' knowledge-sharing behaviors, motivations for knowledge-sharing, and physicians' online seniority status. Then follows a description of the theoretical structure and relationships. Next, we discuss the research methodology and data analysis process. Finally, we present the main findings, implications for theory and practice, and conclusions of the study.

## Literature review

### Physicians' knowledge-sharing in OHCs

Knowledge-sharing in OHCs is defined as the transferability of knowledge among the key participants (e.g., physicians and patients) (23). Knowledge-sharing can generally be described as taking one of two forms: general (public) knowledge-sharing and specific (private) knowledge-sharing (15). Physicians' knowledge-sharing in OHCs could increase their social and economic returns (18) and provide informational and emotional support for patients (24), leading to improved physician–patient relationships (25, 26) and narrowing rural–urban health disparities (3, 27). Most importantly, in physician-driven OHCs, physicians—as crucial sources of general knowledge—attract visits from patients, maintaining the sustainability of the OHC (18).

Extensive studies have explored the antecedents and outcomes of physicians' knowledge-sharing in OHCs from various perspectives. Some scholars found that motivation to help, self-efficacy, moral obligation, and reputation directly and indirectly (through satisfaction) influence health professionals' willingness to continue knowledge-sharing to OHCs (21, 28). Moreover, Yan et al. combined social exchange theory and Maslow's hierarchy of needs theory to analyze that professional users can receive tangible rewards (e.g., bounties, gifts, etc.) and intangible rewards (e.g., reputation, self-esteem, etc.), both of which positively affect their knowledge-sharing behaviors while sharing costs weaken their sharing intentions (15). Similarly, Zhang et al. demonstrated that physicians' professional and material motivations were important in predicting their free health information-sharing in OHCs (17). In addition, Meng et al. indicated that physicians' specific knowledge-sharing behaviors are determined by their general knowledge-sharing behaviors through online reputation (5).

Although studies on knowledge-sharing in the context of OHCs are abundant, the existing literature neglected to explore physicians' continuous knowledge-sharing behaviors from the perspective of the sustainability of OHCs. Physicians' continuous knowledge-sharing are valuable assets to OHCs, and motivating them to continuously share knowledge is critical to the eventual success of OHCs (29). This study has sought to fill this research gap by developing a theoretical framework to explore physicians' motivation to continue sharing knowledge in OHCs and to further examine whether physician motivation is related to their online seniority status. The next section presents a review of the literature on motivation theory and physicians' online seniority status.

### Motivation theory

Motivation is an essential topic of research because it shapes individual behaviors and can be used to understand the types of activities an individual will engage in. Motivation defines the energization and direction of humans to conduct a certain behavior and prompts people to behave in a specific manner (30). In the literature, motivation has usually been divided into extrinsic motivation and intrinsic motivation (31). The former focuses on goal orientation and sees the individual participating in the activity to obtain valuable results, while the latter focuses on the activity itself and portrays the individual as participating in the activity to feel happy and satisfied (32).

Previous research has shown that extrinsic motivation (practical benefits) and intrinsic motivation (psychological rewards) play an essential role in knowledge-sharing. Specifically, practical benefits, a classic extrinsic motivation, reflects the financial or non-financial profits paid to individuals for their effort (18, 33). Psychological rewards, to some degree as intrinsic motivation, pertain to the joy and enjoyment that individuals experience when contributing knowledge to others (34). Similarly, Chang et al. found that reputation can motivate people to participate in knowledge-sharing activities (35). Knowledge contributors who enjoy helping others are more inclined to share knowledge because of the enjoyment and satisfaction they obtain from such behaviors (36). However, some scholars propose a new dimension to explain motivation of knowledge-sharing that is individuals' knowledge-sharing to the community are driven by their concern for the community rather than for self-interest (37). In other words, when members have a strong perceived connectedness to the community, they develop a sense of belonging to the community (38). Previous studies have confirmed that when people have established a sense of belonging to their community, they will take action to avoid losing it (39, 40). When people consider themselves part of the community, they will choose to stay and try to contribute to the community even when unsatisfied with the quality or price of community services. However, in the existing literature, we found little knowledge regarding the motivational effect of physicians' perceived connectedness on their continuous knowledge-sharing behaviors in the context of OHCs.

Based on the above discussion, to provide a holistic view of the motivations behind physicians' continuous knowledge-sharing behaviors, we explored the combined effect of the practical benefits [e.g., financial incentives (12) and online reputation (41)], the psychological rewards [e.g., enjoyment in helping others (14) and psychological satisfaction (13, 17)], and the perceived connectedness with the OHC [e.g., a sense of belonging (19)] in motivating physicians' continuous knowledge-sharing behaviors.

### Physicians' online seniority status in OHCs

The online seniority status of physicians in OHCs refers primarily to the honorary designation that physicians receive in OHCs (22), which reflects the quality of information, timeliness of sharing, and attitude in the process of knowledge-sharing between physicians and patients (42). Physicians with high recognition tend to be those who are more competent, provide better service, and are more popular, and they tend to attract more patients (43) and, consequently, receive more rewards (22). The online seniority status of physicians can also reflect measures of the physicians' expertise and popularity on the platform, especially the level of service and patient satisfaction (44, 45). Hence, a high rating or award for good doctors online is thought to provide a good indicator. Several studies have evaluated the impact of a physician's online reputation on patient choice in the past (42). In examining the moderators of the relationship between motivational factors and physicians' knowledge-sharing behaviors, some past research has mainly focused on the moderating role of the professional status (offline seniority status) of physicians (13). The moderating role of physicians' online seniority status on physicians' continuous knowledge-sharing behaviors still remain largely unknown.

## Research model and hypotheses

**Figure 1:**
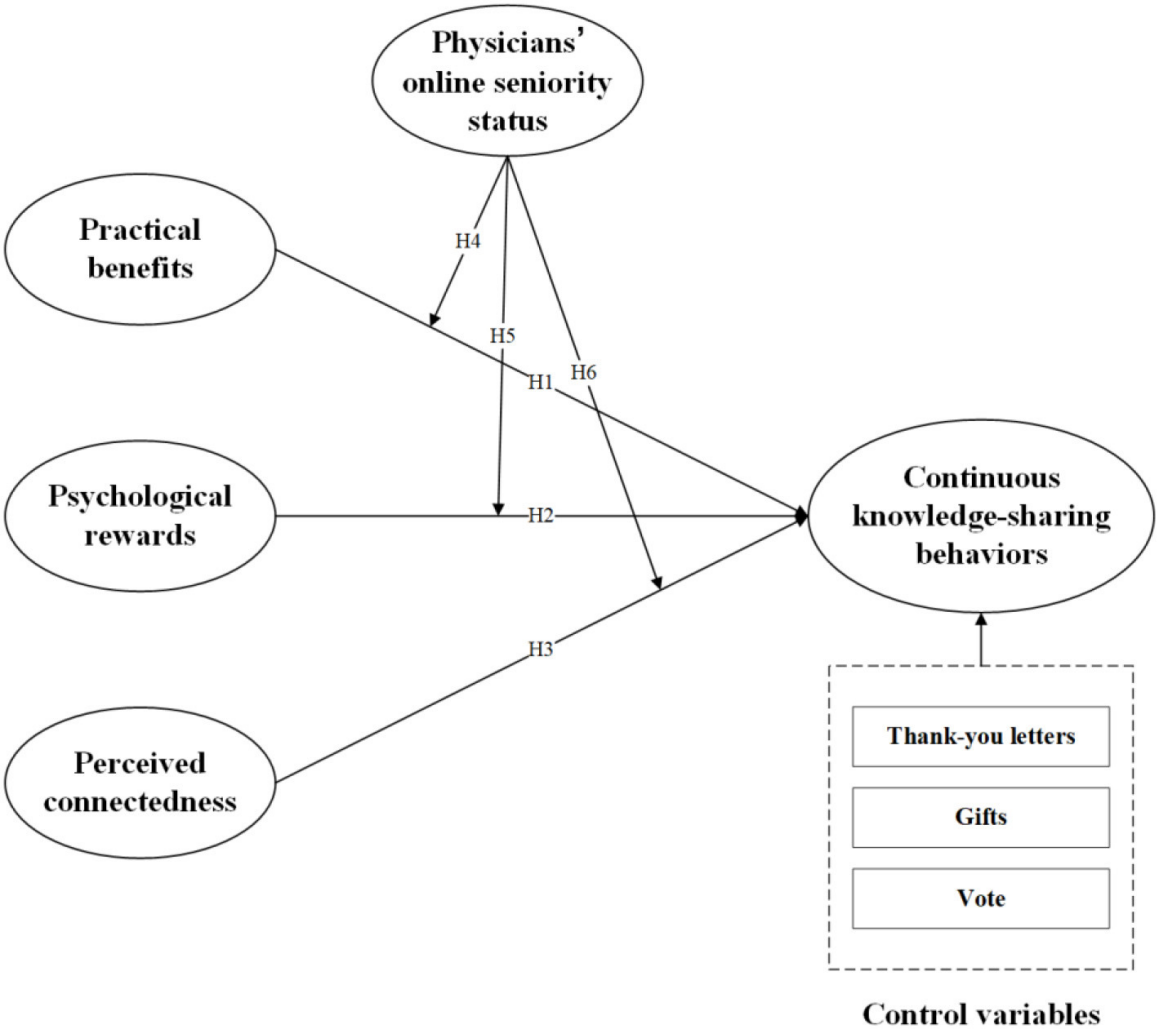
Research model.

To address the issues raised in the above discussion, we developed a theoretical model for explaining continuous knowledge-sharing in OHCs based on the motivation theory (Figure 1). We use physicians' continuous knowledge-sharing behaviors as the dependent variable and practical benefits, psychological rewards, and perceived connectedness as the independent variables. Practical benefits are primarily the financial rewards and reputation that physicians receive from OHCs. Meanwhile, we introduced physicians' online seniority status as a moderating variable in the model.

### The effect of practical benefits on physicians' continuous knowledge-sharing behaviors

Physicians' continuous knowledge-sharing refers to the continuous sharing of knowledge by physicians with their patients (46). Numerous factors have been shown to influence physicians' continuous knowledge-sharing behaviors in OHCs, among which extrinsic motivation relates to the activity as a means to obtain practical benefits (31). Specifically, physicians can obtain monetary rewards through paid counseling services and digital gifts purchased by patients (5). These monetary rewards can provide compensation for physicians' time, effort, and costs in the process of contributing knowledge to OHCs (13). This finding means that physicians who are willing to devote more time and energy to contributing knowledge online will end up earning more online revenue (47). In addition to monetary rewards, reputation, one of practical benefits, has been found to have a positive impact on the knowledge-sharing behaviors of community members (15, 31, 48). In particular, for physicians, reputation is an essential incentive for knowledge-sharing behaviors (16, 21). Therefore, we propose that obtaining more practical benefits in OHCs could incentivize physicians' continuous knowledge-sharing behaviors. Hence, we hypothesized that:

H1: Practical benefits are positively associated with physicians' continuous knowledge-sharing behaviors.

### The effect of psychological rewards on physicians' continuous knowledge-sharing behaviors

Factors that influence physicians' continuous knowledge-sharing behaviors in OHCs also include intrinsic motivation, which generally refers to the psychological rewards that arise from participating in an activity (32, 36). Individuals can gain inner happiness from helping others, which can significantly affect their attitude toward knowledge-sharing (36). In this light, Kankanhalli et al. reported that altruism essentially encourages members to contribute knowledge to the community (31). Accordingly, physicians who are pursuing psychological rewards will actively share their knowledge with patients through providing medical knowledge, treatment experience, and personal advices. By doing so, physicians can gain a sense of being needed, realize their value, and experience feelings of satisfaction regarding their contribution to the online community (36, 49). In this context, physicians can be motivated to continuously sharing knowledge in OHCs by their anticipation and experience of psychological rewards. Therefore, we proposed that psychological rewards could increase physicians' willingness to continue sharing knowledge. Hence, we propose the following hypothesis:

H2: Psychological rewards are positively associated with physicians' continuous knowledge-sharing behaviors.

### The effect of perceived connectedness on physicians' continuous knowledge-sharing behaviors

Perceived connectedness to OHCs refers to the social relationships established with community members; specifically, this connectedness can be described as a sense of belonging and an emotional connection to other members (50). In the context of virtual communities, demand satisfaction is the precursor of virtual community awareness, and emotional connection is highly related to the members (51). Belonging, in this context, is drawn from the need-to-belong theory, which highlights that individuals have a strong desire to establish and maintain close, lasting relationships with others (52, 53). According to the need attribution theory, the motivation to maintain a sense of belonging tends to affect people's engagement (54). This idea suggests that physicians' increased perceived connectedness to OHCs also motivates them to sustain their knowledge-sharing behaviors. In other words, physicians who feel a strong sense of belonging to OHCs gain emotional meaning and self-worth and are therefore more likely to contribute to OHCs (55). Based on the previous discussion, we proposed that physicians' perceived connectedness would have a positive impact on their continued knowledge-sharing behaviors. Based on these arguments, we hypothesized that:

H3: Perceived connectedness is positively associated with physicians' continuous knowledge-sharing behaviors.

### The moderating effects of physicians' online seniority status

A physician's online seniority status in OHCs, according to the status characteristics theory (56), reflects the degree of physician's expertise and contribution to the platform (22). Furthermore, physicians with higher online seniority status possess more economic and social returns compared to those with low online seniority status (18, 57), thus their motivations of contribution in OHCs will be various. Yang et al. demonstrated that when physicians are with higher professional status, their motivations to contributing to OHCs are strengthened by reputation but weakened by monetary rewards (13). According to Maslow's hierarchy of needs theory (58), lower-level needs are generally associated with material factors that are generally reflected in financial status (59). However, when lower-level needs are met, individuals will seek to satisfy higher-level needs that are emotionally or spiritually related, such as love, belonging, and respect (15).

Accordingly, in the context of our study, since physicians with a lower online seniority status in OHCs receive fewer financial incentives from those platforms, practical benefits (e.g., monetary rewards) have a stronger positive effect on physicians who have a low online seniority status than on those with high online seniority status (60). In other words, senior physicians' contribution behaviors in OHCs are prone to be voluntary rather than be motivated by practical benefits. Therefore, we proposed that the positive relationship between practical benefits and physicians' continuous knowledge-sharing behaviors would be weakened when physicians' online seniority status is higher. Based on the above statement, we propose the following hypothesis:

H4: Physicians' online seniority status has a negative moderating effect on the relationship between the practical benefits and physicians' continuous knowledge-sharing behaviors.

Different from practical benefits which focus on economic and social returns, psychological rewards and perceived connectedness are tightly associated with physicians' social acceptance and recognition that satisfy the their emotional or spiritual needs (35). On the one hand, in the condition of higher level of online seniority status, physicians are more concerned about their reputation and patients' positive reviews when they continue sharing knowledge in OHCs (13). Therefore, the positive effect of psychological rewards on physicians' continuous knowledge-sharing behaviors will be improved when physicians are of higher seniority status. On the other hand, physicians with higher online seniority status may perceive a higher responsibility for patients and OHCs, thus motivating them continuing contributing knowledge to OHCs with the aim of maintain or increasing their status. The role of sense of belongingness on physicians' engagement in OHCs can be magnified when they enjoy a higher level of seniority status. Therefore, we proposed that physicians' higher online seniority status will strengthen the effects of psychological rewards and perceived connectedness on physicians' continuous knowledge-sharing behaviors. We accordingly propose the following hypotheses:

H5: The physician's online seniority status has a positive moderating effect on the relationship between psychological rewards and the physician's continuous knowledge-sharing behaviors.

H6: The physician's online seniority status has a positive moderating effect on the relationship between perceived connectedness and the physician's continuous knowledge-sharing behaviors.

## Methodology

### Data and variables

We drew from the “Good Doctor Online” website (www.haodf.com), a leading OHC website in China, as the background of our study. Currently, this website features 891,609 doctors from 10,148 regular hospitals in China. The platform provides an ideal environment for exploring online knowledge-sharing between physicians and patients for the following reasons. First, it enables physicians to share knowledge both publicly (without compensation) and privately (with compensation). Second, it attracts many patients visits and inquiries, which can motivate physicians to share knowledge on the platform. Third, as a leading online health platform, it has a large number of participants, which can provide rich data about physicians' homepages and physician-patient interactions.

Through this platform, patients can submit inquiries to specific physicians to obtain health and medical information about their conditions. Physicians can provide links to their home page, which provides basic information (e.g., hospital, professional title, online contributions, area of expertise, and personal website data statistics) as well as a section in which they can share free health education articles. By doing so, patients can obtain the information they need to learn more about their disease by accessing articles shared by their doctors. If an article matches their preference, they can choose a paid consultation to receive personalized treatment.

We collected data of physicians from haodf.com using a Java-based crawler. We collected data of article publishing and home page information for approximately 80,000 physicians over 6 months (February 2017–July 2017). After removing some samples with incomplete data, we obtained a total number of 308,481 observations. Data were organized in the panel at the monthly level. Table 1 provides descriptive statistics of the study variables. In this study, the dependent variable is physicians' continuous knowledge-sharing behaviors in OHCs, which is measured by the number of new health papers that physicians shared, following the procedures reported in studies by Kuang et al. (61) and Zhang et al. (17). This choice was made because new publications by physicians are seen as a form of continued input into OHCs, and most of these papers are free and open to the public (42, 62, 63). While some studies have used the number of free answers to questions posed by patients to measure physicians' knowledge-sharing behaviors (61), these answers were not directed to the public but to specific groups of patients. For example, a patient with thyroid disease might ask her physician for advice on how to adjust her medication. The doctor subsequently shares answers that can be seen by other visitors; however, the shared information is not specific to these other patients due to their different conditions. In contrast, the free health articles shared by the doctor comprise open access patient education articles, such as descriptions of effective and practical dietary treatments for thyroid disease, which are scientifically accessible to the general reader. Therefore, the number of new responses to questions by physicians was not appropriate in this study for measuring continuous knowledge-sharing behaviors.

**Table 1:** Overview of variables.

**Variables**	**Description**	**Mean**	**SD**	**Min**	**Max**
Newpapers	Number of new papers	6.592432	200.6461	0	53255
Newvisitors	Number of new visitors	212920.2	1210312	3	9.02e+07
Newpatient	Number of new patients	262.1878	1070.949	0	66825
Patientrevisit	Number of patient revisit	15.55301	39.74921	0	1179
Title	Honorary titles for physicians	2.751197	0.976704	1	4
Thank-you	Number of thank-you letters from patients	3.477274	13.75543	0	468
Gift	Number of Online gifts from patients	12.38782	69.23484	0	3060
Vote	Number of votes received by the physician	11.51708	34.85173	0	1179

In our research model, the independent variables were the practical benefits received by the physician, the psychological rewards, and the physician's perceived connectedness. In this OHC, physicians are primarily paid in monetary form for their online services (18). Monetary incentives can be provided to physicians based on one-on-one paid consultations; specifically, physicians receive financial compensation on the platform featured in this investigation for patient consultations. The number of patients highlights the competence of the physician while boosting the physician's reputation at the same time. Therefore, we used the number of new patients as a measure of the practical benefits that physicians receive in OHCs (17). We also took the number of new visitors as an indicator of the psychological rewards that physicians receive to capture their intrinsic motivation. This choice was made because, in addition to practical benefits, physicians have their own intrinsic goals, such as influencing more patients and gaining a high level of patient recognition (64, 65). Patients frequently visit the physicians' home pages indicating that patients are seeking medical knowledge and help from the articles shared by the physicians (66). Although some studies have also used the number of thank-you letters to measure the psychological rewards received by physicians (13, 67) thank-you letters can often only be sent by the physician's patients. However, regular visitors are not able to express their appreciation to the physician through thank-you letters. Therefore, we chose the number of new visitors as a more appropriate measure of psychological reward than the other variables. Meanwhile, the perceived connection of physicians was measured by the number of patient revisits. More such visits would indicate that the physician has a stronger connection to the community and a greater sense of belonging to the community (35). In addition, the number of patient return visits can also reflect the level of doctor–patient interaction (13).

Lastly, the moderating variable in our study model was the online seniority status of the physicians, which was mainly reflected by the physicians' online prestige, in line with the findings of previous studies that the online honorary titles earned by physicians could reflect their prestige (13, 44). Therefore, we used the honorary title earned by the physician in the OHC as a proxy for the physician's online seniority status. Specifically, a physician's honorary title signifies the extent of the patient's recognition of that medical professional in the context of an OHC as a proxy for the physician's expertise, experience, and quality of service. Therefore, the ranking of a physician's honorary title can effectively reflect the physician's online seniority status. We ranked physicians' honorary designations and assigned points to each from highest to lowest.

To ensure the model had a high level of precision, this paper included control variables as follows. Seniority was measured by the professional title of the physician. The gift was measured by the number of online gifts from patients. Thank-you was measured by the number of online thank-you letters from patients. The vote was measured by the number of votes received by the physician.

### Model estimation

To verify the hypothesis that the number of patient revisits, the number of new patients and the number of new visitors would affect the number of new papers published by physicians, and physicians who had higher-level professional titles had a regulatory effect on these factors, this paper developed the following empirical model:


$$\begin{array}{l} \text{newpaper}\;=\;{\text{β}}_{0}\;+\;{\text{β}}_{1}\;\text{newvisit}\;+{\text{β}}_{2}\;\text{newpatient}\;\\ \;+{\text{β}}_{3}\;\text{patientrevisit}\;+{\text{β}}_{4}\;\text{newvisi}{\text{t}}^{*}\text{title}\;\\ \;+\;{\text{β}}_{5}\;\text{newpatien}{\text{t}}^{*}\text{title}\;+{\text{β}}_{6}\;\text{patientrevisi}{\text{t}}^{*}\text{title}\\ \;+\;{\text{β}}^{\prime }\;\text{Z} \end{array}$$

where β is the coefficient, and *Z* is the variable that controls new visitors, new patients, and patient revisits. The model tested the moderating effect of physicians with higher professional titles on the relationship between the number of patients who visited again, the number of new patients, the number of new visitors of patients, and the number of new papers published by the physicians.

The model was tested hierarchically using fixed-effects models. Model, without the moderator variable, was tested to verify the effect of the number of new patients, the number of new visitors, and the number of patient return visits on the number of new papers published by physicians. In Stage 2, Model interaction terms were tested to verify their moderating effects. We used a fixed-effects model to control potential unobserved physician-level heterogeneities.

## Results

For this paper, a fixed effect model was used to test the model. In the first stage, the model was tested without adjusting variables to verify the impact of the number of patients who visited again, the number of new patients, and the number of new visitors of patients on the number of new papers published by physicians. The second stage involved testing the model with interactive items to verify its adjustment effect. In addition, this paper used a fixed effect model to control the potential unobserved heterogeneity at the physician level. Table 2 displays the results.

**Table 2:** Hierarchical regression results.

	**Stage 1**	**Stage 2**
	Newpaper	Newpaper
Patientrevisit	−0.00468***	−0.0589***
	(−12.59)	(−31.13)
Newpatient	0.00315***	−0.0268***
	(12.11)	(−34.75)
Newvisitor	1.00e-05***	0.000204***
	(53.40)	(114.4)
Title	0.00114	0.0208
	(0.0708)	(1.268)
Newvisitor*title		−4.98e-05***
		(−109.4)
Newpatient*title		0.00767***
		(29.57)
Patientrevisit*title		0.0139***
		(26.63)
Constant	0.0872*	−9.40e-05
	(1.767)	(−0.00185)
Observations	131,849	131,849
R-squared	0.024	0.106
Adjusted *R*^2^	0.106	0.106
*F*-test	0	0

Standard errors are in parentheses.

^***^p < 0.01, ^*^p < 0.1. All levels of significance are higher than *p* < 0.1.

Hypothesis 1 proposed that practical benefits are positively associated with physicians' continuous knowledge-sharing behaviors. According to the data in the first column of Table 2, this hypothesis was supported because the coefficient of new patients (β = 0.00315, *t* = 12.11, *p* < 0.01) was positive and statistically significant.

Hypothesis 2 proposed that psychological rewards would be positively associated with physicians' continuous knowledge-sharing behaviors. The data in the first column of Table 2 reveal that this hypothesis was supported because the coefficient of the new visitors (β = 1.00e-05, *t* = 53.40, *p* < 0.01) was positive and statistically significant.

Hypothesis 3 proposed that the perceived connectedness would be positively associated with the physicians' continuous knowledge-sharing behaviors. However, the data in the first column of Table 2 indicate that this hypothesis was not supported because the coefficient of the patient revisits (β = 1.00e-05, *t* = 53.40, *p* < 0.01) was negative and statistically significant.

In the second stage, the moderating effect of the physician's online seniority status was tested, and we found evidence to support Hypothesis 6. According to the data in the second column of Table 2, the coefficient of the interaction term patientrevisit^*^title (β = 0.0139, *t* = 26.63, *p* < 0.01) was positive and significant. However, Hypotheses 4 and 5 were not supported because the coefficient of the interaction term newpatient^*^title (β = 0.00767, *t* = 29.57, *p* < 0.01) was positive and statistically significant, while the coefficient of the interaction term newvisitor^*^title (β = −4.98e-05, *t* = −109.4, *p* < 0.01) was negative and significant.

### Robustness check

To test the robustness of our model, following the suggestion of previous studies (13), we extracted a subsample of the total sample and ran the model again. First, to ensure that the findings of our empirical analysis are not influenced by a specific population, the subsample selected for this paper is doctors who have been registered with the “Good Doctor Online” website for a long time and have been online recently. Then, we extracted data from these doctors and selected a total of 5,136 data. Finally, we reran the model with this set of data to test our hypothesis. The results of the robustness test revealed that the coefficients of new patients (β = 0.0765, *t* = 6.85, *p* < 0.01) and new visitors (β = 3.95e-06, *t* = 2.72, *p* < 0.01) were still positive and significant, while the coefficients of patient return visits (β = −0.0041, *t* −3.81, *p* < 0.01) were negative and significant. In the second stage of the test, the coefficients of interaction terms newpatient^*^title (β = 0.00016, *t* = 2.96, *p* < 0.01) and patientrevisit^*^title (β = 0.00073, *t* = 2.55, *p* < 0.05) were still positive and significant, and the coefficients of interaction term newvisitor^*^title (β = −9.79e-07, *t* = −2.68, *p* < 0.01) were negative and significant. This result was consistent with the previous results. Therefore, we could be more confident that the results of our analysis were solid and robust.

## Discussion and implications

### Discussion

This paper explored the motivational factors that influence physicians' continuous knowledge-sharing in the OHC and the moderating role of physicians' online honorary titles. We hypothesized that in the context of an OHC, the practical benefits, the psychological rewards, and the perceived connectedness with the community would have an influential role on physicians' continuous knowledge-sharing behaviors on the platform. In addition, we hypothesized that physicians' online honors would have a moderating effect on these influences. Several key findings emerged in this investigation.

First, the practical benefits of an OHC were positively associated with physicians' continuous knowledge-sharing behaviors in OHCs. This result is consistent with previous findings in other contexts (12, 15, 68) that reputation as well as monetary rewards can motivate physicians to contribute to OHCs. Thus, based on motivation theory our results again validate the motivating effect of extrinsic motivation on physicians' continuous knowledge-sharing behaviors.

Second, our study found that psychological rewards were positively associated with physicians' continuous knowledge-sharing behaviors in the OHC and that psychological rewards received by physicians significantly influenced their continuous knowledge-sharing behaviors. Consistent with previous findings (12, 15), psychological rewards can motivate physicians' continuous knowledge-sharing behaviors in an OHC. The knowledge shared by physicians helps more people, and physicians can gain more psychological satisfaction, which in turn motivates them to continue to share knowledge. New visitors can also serve as potential patients for the physician, affording the possibility of giving the physician extrinsic rewards in the future by paying him/her for consultations.

Third, we found that the perceived connectedness with the OHC negatively influenced physicians' continuous knowledge-sharing in the OHC, which differed from the results of previous studies (13). A possible explanation for this unsupported hypothesis is that the analysis of the physician–community connection in this research was primarily between physicians and past patients who were often cured after treatment by physicians. Physicians have limited energy, and to help more patients, they tend to focus more on existing patients and will spend less time and effort on past patients. On the other hand, physicians attract more patients by publishing free articles online, and patients often go from online to offline, visiting the doctor's hospital, and establishing an offline connection with the physician so that there are no more late revisits (5). Moreover, patient return visits do not bring financial rewards to physicians, and physicians are not compensated for the cost of their time, thus negatively impacting physicians' knowledge-sharing behaviors.

Fourth, we found that the online seniority status plays an essential moderating role in the relationship between motivational factors and sustained knowledge-sharing among physicians. Although both practical benefits and psychological rewards can have a positive effect on physicians' continuous knowledge-sharing behaviors, the two motivational factors have different strengths for physicians with different online seniority statuses. The higher the online seniority status of physicians in OHCs, the stronger the role of practical benefits and the weaker the role of psychological rewards. And previous research on the moderating effect of physicians' offline professional titles found that the higher the physician's offline title, the lower the incentive for practical benefits (13). The reason for this different result is that many physicians who do not have high offline professional titles may have achieved high titles through their efforts in the OHC. The main source of income for physicians is their salaries from offline work, suggesting that although many physicians have high online seniority status, their actual economic reward is still limited. Many physicians with low offline professional titles actively contribute to the OHC, and these physicians often hope to gain more reputation in the OHC to increase their influence and gain more economic rewards to improve their economic situation. Physicians with higher online seniority status, are more likely to attract several patients for paid consultations. Their existing patient base is much larger than that of physicians with lower online credential status. Since physicians have limited energy, those with higher online status tend to focus more on existing patients rather than new visitors. In addition, we found that physicians' online seniority status weakened the negative relationship between perceived connectedness and physicians' continuous knowledge-sharing behaviors. Physicians with high online seniority status cared more about community affiliation than physicians with low online seniority status. A possible explanation for this result is that physicians with higher online seniority status tend to work harder to maintain their connection with patients in the community to maintain their already honored titles. The results of this study reveal the moderating mechanism of physicians' online seniority status in the relationship between motivational factors and physicians' continuous knowledge-sharing behaviors.

### Theoretical implications

Our research contributes to the literature on knowledge-sharing and OHCs from several perspectives. First, this study is one of the first to investigate physicians' continuous knowledge-sharing behaviors from the sustainable development perspective of OHCs by exploring how practical benefits, psychological rewards, and perceived connectedness jointly influence continuous knowledge-sharing behaviors. Although previous research has explored physicians' motivations for knowledge contribution in OHCs, most were limited to exploring the motivations for physicians' knowledge-sharing behaviors (12, 21, 28), and lack of understanding of the motivations for physicians' continuous knowledge-sharing behaviors in the long run. Accordingly, this study addressed this research gap in knowledge-sharing in OHCs by revealing motivational factors of physicians' continuous knowledge-sharing behaviors.

Second, this study complements motivation theory in the context of knowledge-sharing in OHCs by specifically incorporating physicians' perceived connectedness into the motivational model to predict physicians' continuous knowledge-sharing behaviors. Previous research has extended the understanding the role of extrinsic and intrinsic motivation on knowledge-sharing behaviors in OHCs. However, most studies lack of considering the social attributes of OHCs (37, 38), therefore may fail to provide a comprehensive view regarding the motivation of physicians' continuous knowledge-sharing behaviors in OHCs. To fill this research gap, this study represents the first attempt to examine the combined effects of practical benefits, psychological rewards, and perceived connectedness on physicians' continuous knowledge-sharing behaviors.

Finally, in contrast to prior studies concerning OHCs focusing solely on the role of physicians' offline seniority status (13, 69), this study tested the moderating role of physicians' online seniority status (honorary title in OHCs) on their continuous knowledge-sharing behaviors. The findings shed the light on the role of online seniority status which is as important as offline seniority status in motivating physicians' knowledge-sharing behaviors in OHCs.

### Practical implications

Our study provides several important implications for practitioners of OHCs. First, our findings indicate that both practical benefits and psychological rewards positively influence physicians' continuous knowledge-sharing behaviors. In other words, physicians can continuously benefit from their knowledge-sharing in OHCs, such as tangible benefits and psychological rewards. Therefore, to address the problem of physicians' inadequate contributions and sustainable development of OHCs, the platform managers should emphasize the practical benefits and psychological rewards of continuous knowledge-sharing for physicians with various levels of online seniority, thus better motivating physicians to share knowledge in OHCs. Specifically, the platform should increase practical benefits for physicians with low online seniority and strengthen psychological rewards for physicians with high online seniority, respectively, to motivate all physicians' continuous knowledge-sharing behaviors.

Second, inconsistent with our hypothesis, perceived connectedness is negatively associated with physicians' continuous knowledge-sharing behaviors. In other words, a large number of patients revisiting physicians' homepages (more potential patients) will lower physicians' motivation to continue sharing knowledge in OHCs. To weaken the negative effect of perceived connectedness on physicians' continuous knowledge-sharing behaviors, some measures should be taken such as lowering the threshold of interaction, optimizing the product design, simplifying the operation of patient–physician interaction, or making the act of interaction more interesting. In addition, managers of OHCs can provide physicians extra monetary rewards when the number of visitors (new visitors and returning visitors) to their homepages reaches a certain level to cover the cost of physician engagement.

### Limitations and future research

This study has some limitations. First, although we examined the moderating effect of physicians' online seniority status on the relationship between incentives and physician behavior, the strength of the moderating effect could be influenced by other characteristics of physicians. For example, physicians' demographic characteristics and medical specialties may influence the moderating effect of online rank. In future research, we intend to explore the role of physician characteristics in our research model with multiple considerations.

Second, our study focused on the effect of online incentives on physicians' knowledge-sharing behaviors in OHCs. However, physicians' online behavior can be influenced by offline behavior. Usually, physicians use their time outside of work to provide services on an OHC; moreover, the rules and regulations of the hospitals where physicians work also have an impact on physicians' online behavior. That said, obtaining data on physicians' offline behaviors to study the influence of offline factors on physicians' knowledge-sharing behaviors in OHC entails various difficulties. In future studies, offline motivational factors should be included in the research model.

Finally, in this paper, only physicians' data from the “Good Doctor Online” website was collected. It is necessary to collect more extensive data from other platforms simultaneously to further validate our hypothesis. Therefore, data from several more OHCs should be collected in future research endeavors to explore any gaps that may exist between different platforms.

## Conclusion

Physicians' continuous knowledge-sharing behaviors are crucial for the sustainable development of OHCs as well as patient education. Therefore, how to motivate physicians to continuously share their knowledge in OHCs has drawn the attention of related scholars. Based on motivation theory, this study constructed an integrated motivational model to explore the motivational factors on physicians' continuous knowledge-sharing behaviors as well as the moderating role of physicians' online seniority status. The research model and relevant hypotheses were tested using real data from the “Good Doctor Online” website. The results indicate that practical benefits and psychological rewards positively influence physicians' knowledge-sharing behaviors. Inconsistent with our hypothesis, perceived connectedness is found to have a negative effect on physicians' continuous knowledge-sharing behaviors. In addition, physicians' online seniority status moderated the relationships between motivational factors (practical benefits, psychological rewards, and perceived connectedness) and continuous knowledge-sharing behaviors. This study makes a vital contribution to the existing literature regarding motivation theory, knowledge-sharing, and OHCs. In practice, our findings provide crucial insights and strategies for designers and practitioners of OHCs to manage and motivate physicians' sustainable contributions.

## Data availability statement

The original contributions presented in the study are included in the article/supplementary files, further inquiries can be directed to the corresponding author/s.

## Author contributions

All authors listed have made a substantial, direct, and intellectual contribution to the work and approved it for publication.

## Funding

This study was funded by the National Natural Science of China (72001094).

## Conflict of interest

The authors declare that the research was conducted in the absence of any commercial or financial relationships that could be construed as a potential conflict of interest.

## Publisher's note

All claims expressed in this article are solely those of the authors and do not necessarily represent those of their affiliated organizations, or those of the publisher, the editors and the reviewers. Any product that may be evaluated in this article, or claim that may be made by its manufacturer, is not guaranteed or endorsed by the publisher.
